# The neurofibromatosis type I gene promotes autophagy via mTORC1 signalling pathway to enhance new bone formation after fracture

**DOI:** 10.1111/jcmm.15767

**Published:** 2020-08-30

**Authors:** Qian Tan, Jiang‐Yan Wu, Yao‐Xi Liu, Kun Liu, Jin Tang, Wei‐Hua Ye, Guang‐Hui Zhu, Hai‐Bo Mei, Ge Yang

**Affiliations:** ^1^ Department of Orthopedic Surgery The Hunan Children's Hospital Changsha China

**Keywords:** autophagy, fracture, osteogenesis, the neurofibromatosis type I gene

## Abstract

Bone fracture is one of the most common injuries. Despite the high regenerative capacity of bones, failure of healing still occurs to near 10% of the patients. Herein, we aim to investigate the modulatory role of neurofibromatosis type I gene (NF1) to osteogenic differentiation of bone marrow–derived mesenchymal stem cells (BMSCs) and new bone formation after fracture in a rat model. We studied the *NF1* gene expression in normal and non‐union bone fracture models. Then, we evaluated how *NF1* overexpression modulated osteogenic differentiation of BMSCs, autophagy activity, mTORC1 signalling and osteoclastic bone resorption by qRT‐PCR, Western blot and immunostaining assays. Finally, we injected lentivirus‐*NF1* (Lv‐*NF1*) to rat non‐union bone fracture model and analysed the bone formation process. The *NF1* gene expression was significantly down‐regulated in non‐union bone fracture group, indicating *NF1* is critical in bone healing process. In the *NF1* overexpressing BMSCs, autophagy activity and osteogenic differentiation were significantly enhanced. Meanwhile, the *NF1* overexpression inhibited mTORC1 signalling and osteoclastic bone resorption. In rat non‐union bone fracture model, the *NF1* overexpression significantly promoted bone formation during fracture healing. In summary, we proved the *NF1* gene is critical in non‐union bone healing, and *NF1* overexpression promoted new bone formation after fracture by enhancing autophagy and inhibiting mTORC1 signalling. Our results may provide a novel therapeutic clue of promoting bone fracture healing.

## 1 INTRODUCTION

Bone fracture healing cascades involve a series of complex biophysiological and pathological processes, which need the participation of various cellular and biochemical cues. Depending on the nature of the injury and the post‐injury care, it can take several months for bone fracture to repair. Although the bone tissue has strong regenerative capacity among the human organs, for certain type of fractures such as tibia diaphysis, or for patients with limited healing capabilities, delayed repair or facture non‐union may happen,^1^ leading to impaired rehabilitation and poor repair quality. Current therapies to address failed fracture repair include non‐surgical and surgical treatments. The most common non‐surgical treatment is to use electrical stimulator; however, with different devices used, such as direct current, inductive coupling and capacitive coupling, the effectiveness may be inconsistent for treating non‐union.^2^ Surgical treatments on the other hand will usually introduce secondary trauma and raise the risk of infection. Therefore, new therapies to enhance bone formation after fracture are in need.

Neurofibromatosis type I (*NF1*) is an inherited autosomal dominant disorder caused by mutation in the tumour suppressor *NF1* gene. This disease affects about 1:3000 world population, and the symptoms include pigmentary lesions, dermal neurofibromas, and in some cases, skeletal abnormalities.^3^ The *NF1* gene encodes neurofibromin 1, which is a Ras‐GTPase activating protein (Ras‐GAP). The neurofibromin has been shown to be essential in osteoblast functioning; therefore, mutation in the *NF1* gene results in skeletal abnormalities such as scoliosis, unilateral growth and congenital pseudoarthroses of long bones.^4^ Moreover, the *NF1* gene expression is also found in adult osteoblasts and osteoclasts, as well as hypertrophic chondrocytes, which presents during endochondral ossification.^5^ The involvement of the *NF1* gene and skeletal development provides a potential therapeutic target to modulate bone fracture healing.

Bone marrow mesenchymal stem cells (BMSCs) are multipotent stem cells that bestow the high regenerative potential of human skeleton. During bone fracture repair, BMSCs are activated and differentiated into osteoblasts, and bone marrow–derived macrophages (BMDM) differentiated into osteoclasts. The balance between bone resorption and formation is critical to fracture repair. Increased activity of osteoclasts will result in higher rate of bone resorption that compromises the repair efficiency. Autophagy is the body's self‐cleaning process that is essential for cell viability, differentiation and metabolism.^6^ It is reported that autophagy plays an important role in bone cell function and pathology.^7^ Specifically, for BMSCs differentiation, emerging evidences proved that autophagy was involved in osteogenic differentiation and bone mineralization.^8^, ^9^ Qiao et al^10^ reported that the *NF1* overexpression induced autophagy and inhibited mTOR/P70S6K signalling in ovarian carcinoma cells and in turn inhibited ovarian carcinoma cell proliferation and invasion. Mammalian target of rapamycin (mTOR) functions critically in the metabolic signalling, proliferation, autophagy and cellular fate.^11^ Park et al^12^ reported that modulating mTOR signalling resulted in different cell fate such as autophagy vs proliferation balance via binding and phosphorylating autophagy‐related gene 14 (ATG14). However, the role of *NF1*/autophagy functioning axis is yet to be investigated in osteogenic differentiation and bone formation.

In our current study, we evaluated the modulatory role of the *NF1* gene on bone formation during fracture repair, and we investigated in depth of the possible mechanisms. We found the *NF1* gene was able to enhance autophagy via inhibiting mTOR complex 1 (mTORC1) signalling, leading to pro‐osteogenic and anti‐osteoclastic differentiation. Our study demonstrated *NF1* may be one of the critical therapeutic targets to promote bone fracture healing.

## 2 MATERIALS AND METHODS

### 2.1 BMSCs isolation and culture

Bone marrow mesenchymal stem cells were isolated from Sprague‐Dawley (SD) rat femur. In brief, the bone marrow was flushed using a disposable aseptic syringe with antibiotic supplemented phosphate‐buffered saline (PBS) (Gibco). The cell suspension was centrifuged at 225 *g* for 5 minutes at room temperature and rinsed in mesenchymal stem cell growth medium (Cyagen Biosciences). Cell pellet was resuspended with mesenchymal stem cell growth medium supplemented with 10% foetal bovine serum (FBS) (Gicbo), 1× (100 IU/mL penicillin and 0.1 mg/mL streptomycin) penicillin‐streptomycin (Pen‐strep) (Life Technologies), and 2 mmol/L glutamine (Life Technologies); and cultured in 5% CO_2_ humidified incubator at 37°C. Osteoblast differentiation was induced with osteogenic medium (Cyagen Biosciences) supplemented with 10% FBS, 1× pen‐strep, 2 mmol/L glutamine, 1 × 10^−4^ mmol/L dexamethasone (Sigma Aldrich), 10 mmol/L β‐glycerophosphate (Sigma Aldrich) and 50 µg/mL L‐ascorbate acid (Sigma Aldrich).

### 2.2 Cell transfection and treatment

Lentivirus vectors of green fluorescent protein (GFP) control and the NF1 overexpression were constructed by Hanbio Biotechnology (Hanbio Biotechnology Co., Ltd). Cells were transfected with different lentivirus vectors for 48 hours after reaching 80% confluency. After transfection, 3 mmol/L autophagy inhibitor 3‐Methyladenine (3‐MA), or 30 µmol/L mTOR activator 3‐Benzyl‐5‐((2‐nitrophenoxy) methyl)‐dihydrofuran‐2 (3H)‐one (3‐BDO) were used to treat cells for 12 and 24 hours, respectively. MEK inhibition treatment was performed using 10 nmol/L PD0325901 (Sigma Aldrich) for 24 hours; then, BMSCs were cultured in osteogenic medium, and BMDMs were cultured in osteoclast differentiation medium. For the in vivo study, the GFP or *NF1* lentivirus vectors were injected to the fracture site with a microsyringe (Hamilton).

### 2.3 Quantitative real‐time polymerase chain reaction (qRT‐PCR)

Total RNA was extracted using TRIzol reagent (Invitrogen) and purified with RNeasy mini kit (Qiagen) following the manufacturer's protocol. Total quantity of 1 µg of RNA was reverse transcript to cDNA with High Capacity cDNA kit (Thermo Fisher Scientific). qRT‐PCR was performed using SYBR Green master mix (Thermo Fisher Scientific), and the relative expression of the *NF1* gene was calculated using the 2^−∆∆^ *^C^* ^t^ method and normalized to GAPDH. The sequence information of primers used in this study was as listed below:

*NF1* (forward): 5′‐GGAATGGCACTGCAAGCAAA‐3′,

*NF1* (reverse): 5′‐GCAACAATGGCAGGTGAAGG‐3′;

GAPDH (forward): 5′‐GCAAGTTCAACGGCACAG‐3′,

GAPDH (reverse): 5′‐GCCAGTAGACTCCACGACAT‐3′.

### 2.4 Western blot

Total proteins from BMSCs or bone tissues were extracted using Radio‐Immunoprecipitation Assay Lysis Buffer (Beyotime). Same amount of protein samples (50 µg) were separated by SDS‐PAGE then transferred to polyvinylidene difluoride (PVDF) membranes. The loaded membranes were blocked and stained with primary antibodies at 4°C overnight. Horseradish peroxidase‐conjugated secondary antibody was used to stain the membranes for 1 hour at room temperature, and then, the samples were visualized with enhanced chemiluminescence detection system (Millipore). The primary antibodies were listed below: GAPDH (1:1000), Osterix, Runt‐related transcription factor 2 (Runx2), alkaline phosphatase (ALP), microtubule‐associated proteins 1A/1B light chain 3B (LC3) II/I, Sequestosome‐1 (p62), Beclin1, phosphorylated mTORC1 (p‐mTORC1), mTORC1, p‐S6K1/S6K1, p‐4EBP1/4EBP1 (1:1000), Osteocalcin (OCN) and *NF1* (1:800). All antibodies were purchased from Abcam. Besides, p‐ERK/ERK (1:1000) were purchased from Cell Signaling Technology.

### 2.5 Flow cytometry

Bone marrow mesenchymal stem cells were cultured in growth medium, and cells from passage 3 were harvested and stained with CD90, CD44, CD34 and CD31. Briefly, cells were digested with 0.05% trypsin and washed with cold PBS. Cell pellets were resuspended in blocking buffer at concentration of 1 × 10^6^ cell/mL. Then, 100 µL of the cell suspension was transferred to a 5 mL culture tube and stained with CD90, CD44, CD34 and CD31 antibodies at concentration of 1:500. All antibodies were purchased from Biolegend. After 2 washes with cold PBS, the stained cells were resuspended in 500 µL PBS and then analysed by flow cytometry.

### 2.6 ALP and Alizarin red staining

Bone marrow mesenchymal stem cells that underwent osteogenic induction were fixed with 4% paraformaldehyde for 10 minutes. The ALP staining was conducted with the BCIP/NBT regent kit (Beyotime), and Alizarin Red staining, cells were stained with Alizarin Red solution (Cyagen Biosciences). All procedures were carried according to the manufacture's protocols.

### 2.7 Immunofluorescence staining

The BMSCs layers were washed with PBS and fixed in 4% paraformaldehyde (PFA) for 15 minutes. The cells were blocked in 1% BSA in PBS for 30 minutes at room temperature and then stained in anti‐p‐mTORC1 (1:250) antibody at 4°C overnight. After staining with secondary antibody for 1 hour at room temperature, the cell layers were washed for 3 times and counterstained DAPI for 10 minutes. Then, samples were imaged with fluorescence microscopy (Zeiss).

### 2.8 Osteoclast differentiation

Bone marrow (BM) cells collected from SD rat femur were cultured in α‐Minimum Essential Medium (α‐MEM) for 3 hours to initiate adherence; then, the non‐adherent cells in the medium were collected and re‐seeded at a density of 1 × 10^6^ cells/mL. The cells were cultured in complete medium with macrophage colony‐stimulating factor (M‐CSF) (20 ng/mL; Sigma Aldrich) for 3 days; then, the medium was refreshed every 3 days with complete medium supplemented with M‐CSF (20 ng/mL) and receptor activator of nuclear factor kappa‐Β ligand (RANKL) (20 ng/mL; Sigma Aldrich).

### 2.9 Tartrate‐resistant acid phosphatase (TRAP) staining and bone resorption

The differentiated osteoclast cells were fixed with 4% PFA and stained with TRAP‐kit (Sigma) according to the manufacturer's instructions. Mature osteoclasts were counterstained with methylene blue. For bone resorption assay, the differentiated osteoclast cells were cultured on the pre‐made dentin discs for 12 days. The resorption pits of the bone slices were imaged with bright field microscope (Zeiss).

### 2.10 Enzyme‐linked immunosorbent assay (ELISA)

The medium of the differentiated osteoclasts was refreshed with serum‐free α‐MEM 24 hours prior to harvest. At harvest, the supernatant was collected and analysed using C‐telopeptide of type I collagen ELISA kit (Biocalvin) for CTX and N‐telopeptide of type I collagen ELISA kit (Biocalvin) for NTX.

### 2.11 Rat model of bone fracture

Healthy male SD rats (around 2 months old) were purchase from SLAC. All the animal experimental procedures were approved by the Animal Experiment Committee of Hunan Children's Hospital (Changsha, China) and performed in accordance with the guidelines and regulations. The rats were anesthetized with an intraperitoneal injection of ketamine, and the middle femoral fracture surgery was performed on the right femur. In brief, we made a transverse incision and carefully removed the skin and subcutaneous tissue to expose the right femoral bone shaft. Fracture was created by a metal‐wire saw. To fix the fracture site, a Kirschner wire was inserted to align the fracture site. Then, the subcutaneous incision was sutured close. In the non‐union group, the periosteum of femur was removed as much as possible. The determinations were performed at 8 weeks post‐surgery.

### 2.12 Histology

The femurs of each group of SD rats were decalcified in 10% ethylenediaminetetraacetic acid (EDTA) (pH 7.0) and embedded in paraffin according to standard protocol. The sectioned slices were stained with haematoxylin and eosin, and Masson's trichrome according to the manufacture's protocols.

### 2.13 Statistics

All experiments were repeated independently for at least three times with n = 6 in each group. Statistical analyses were performed using Prism software (GraphPad Prism 8, USA). One‐way analysis of variance (ANOVA) or Student's *t* test was used to analyse the data, and *P* < .05 was considered statistically significant.

## 3 RESULTS

### 3.1 The NF1 gene expression is inhibited in non‐union model

**FIGURE 1:**
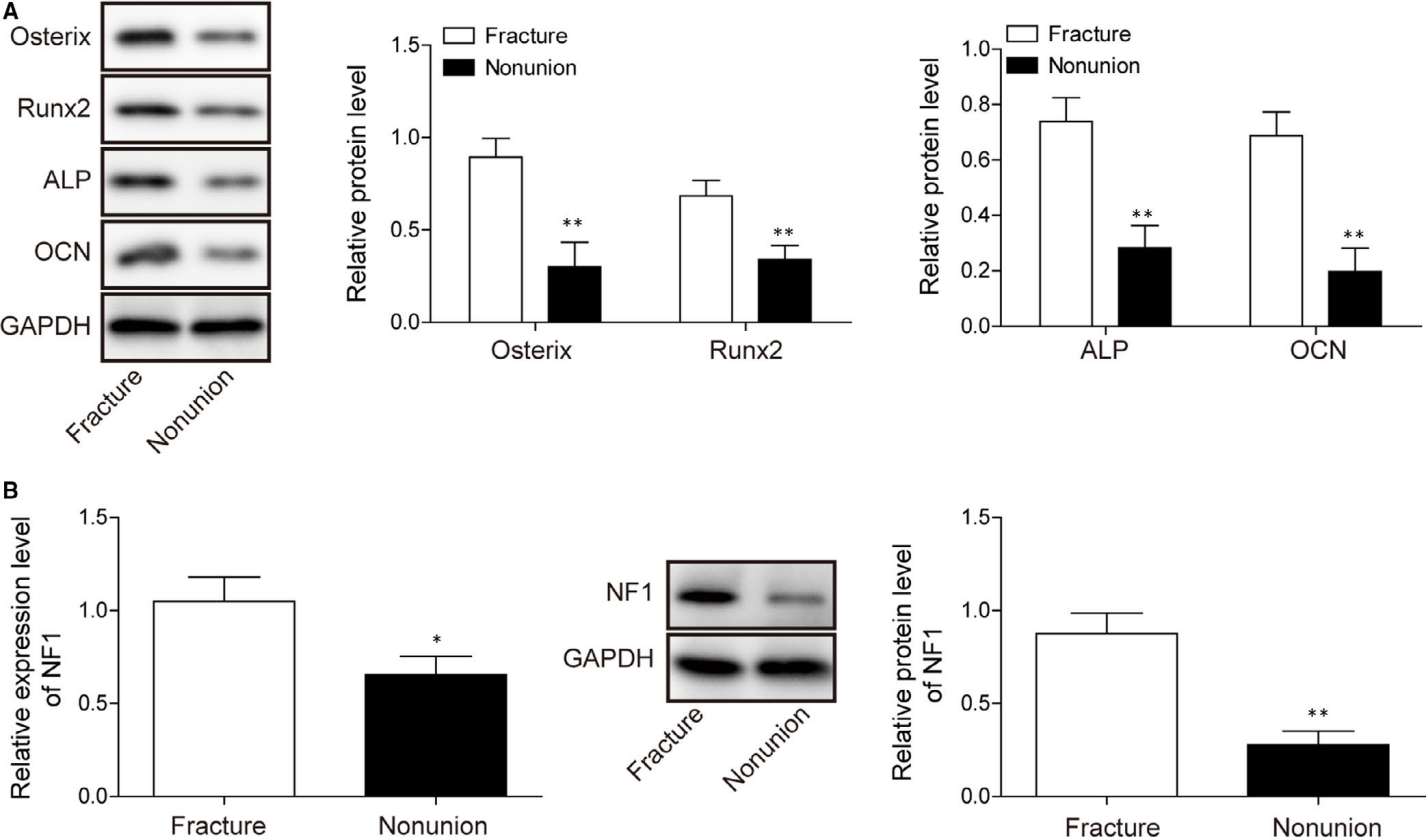
The *NF1* gene expression in normal and non‐union bone fracture model. A, Western blot of osteogenic markers Osterix, Runx2, ALP and OCN and the intensity analysis of normal and non‐union bone fracture model. B, The *NF1* transcript and *NF1* protein level in normal and non‐union bone fracture model. All data were presented as mean ± SD. *P < .05; **P < .01. n = 6 in each group

We established rat bone fracture model to evaluated the difference of normal bone fracture healing from non‐union. Western blot data demonstrated osteogenic protein expressions in the normal fracture and non‐union groups at 4 weeks post‐surgery. In comparison with normal fracture group, non‐union rats showed significantly less osteogenic protein Osterix, Runx2, ALP and OCN expressions, indicating poor osteogenesis (Figure 1A). We found significant difference in the *NF1* gene expression and protein production in normal fracture and non‐union groups (Figure 1B), as non‐union rat expressed significantly lower *NF1* in comparison with normal fracture rats on both transcript and protein levels. Elevated ERK activity was also observed in non‐union bones (Figure S1A). These results demonstrate osteogenesis and the *NF1* gene are both inhibited in non‐union models, indicating potential correlation between *NF1* and osteogenesis.

### 3.2 The NF1 gene enhances osteogenic differentiation via promoting autophagy

**FIGURE 2:**
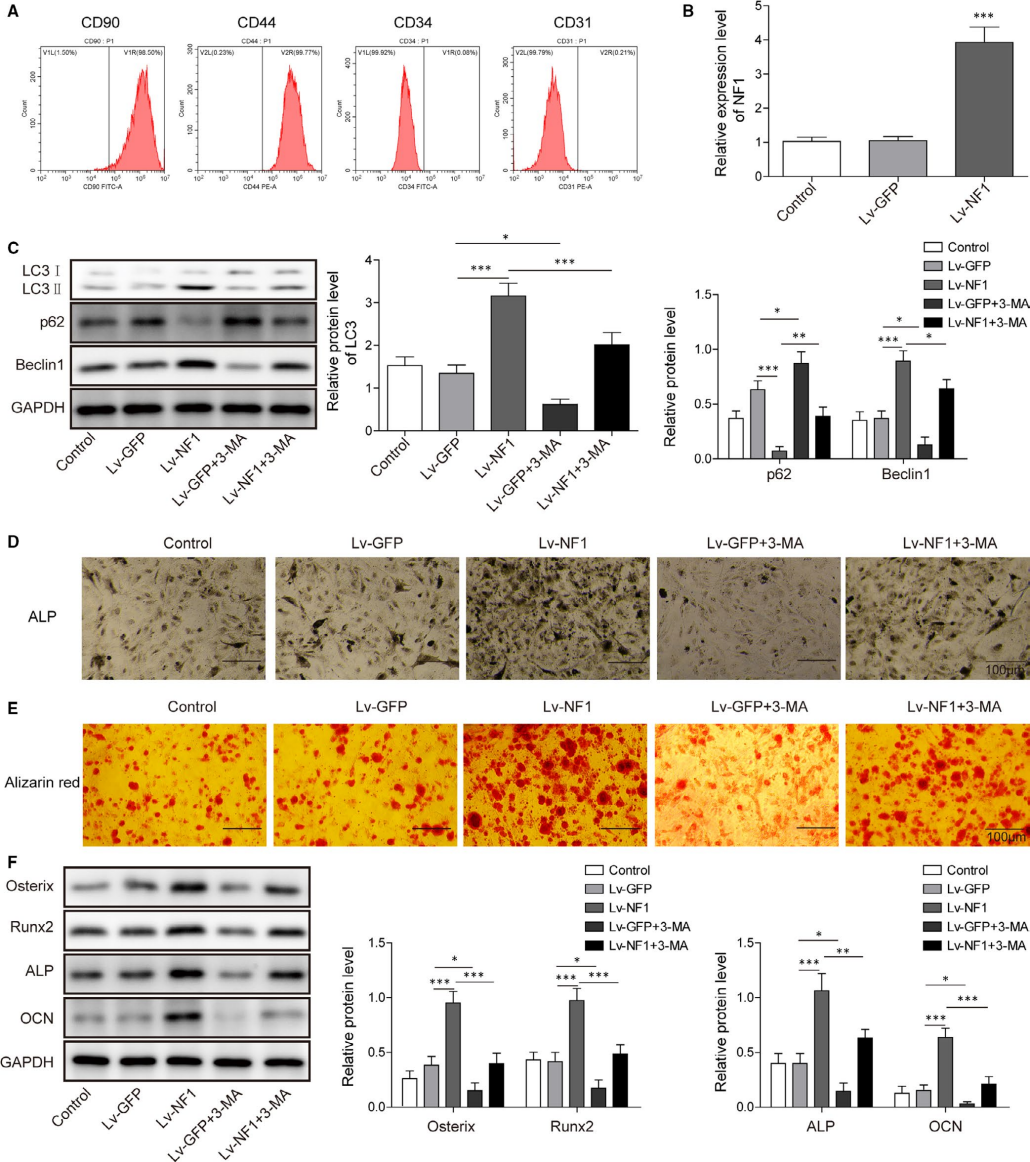
The *NF1* gene activates autophagy and promotes osteogenic differentiation of BMSCs. A, Flow cytometry analysis of CD90, CD44, CD34 and CD31. B, The *NF1* gene expression of BMSCs after transfection. C, Autophagy‐related protein levels of control and transfected BMSCs. D, ALP and (E) Alizarin Red staining of control and transfected BMSCs. F, Western blot of osteogenic markers Osterix, Runx2, ALP and OCN in control and transfected BMSCs with GAPDH as reference gene. All data were presented as mean ± SD. *P < .05; **P < .01; ***P < .001. n = 6 in each group

We next evaluated how *NF1* influenced osteogenic differentiation of BMSCs. Flow cytometry graph presented the isolated cells stained positively in CD90 and CD44, whereas negatively in CD34 and CD31, exhibiting the standard phenotype of BMSCs (Figure 2A). The transfection efficacy of *NF1* overexpression was validated by qRT‐PCR (Figure 2B). *NF1* overexpressing group (Lv‐*NF1*) showed significantly higher *NF1* gene expression as compared to control and Lv‐GFP group, indicating a successful transfection. The autophagy‐related markers LC3II/I, Beclin1 and p62 were evaluated by Western blotting. The *NF1* overexpression enhanced LC3 II/I and Beclin1 expressions, and inhibited p62 expression, indicating an enhancement of autophagy activity. Autophagy inhibitor 3‐MA partially reversed the pro‐autophagic effect of *NF1* overexpression (Figure 2C). The osteogenic differentiation of BMSCs was also impacted by *NF1* overexpression and autophagy inhibition. Osteogenic marker ALP (Figure 2D) and Alizarin Red (Figure 2E) staining showed *NF1* overexpression enhanced both ALP activity and mineralization, while 3‐MA partially suppressed the osteogenic effects induced by *NF1* overexpression. Western blotting data further demonstrated 3‐MA partly reversed the increase of osteogenic protein Osterix, Runx2, ALP and OCN expressions induced by *NF1* overexpression (Figure 2F). All data above indicate *NF1* enhances osteogenic differentiation of BMSCs via promoting autophagy.

### 3.3 The NF1 gene promotes autophagy via inhibiting mTORC1 signalling

**FIGURE 3:**
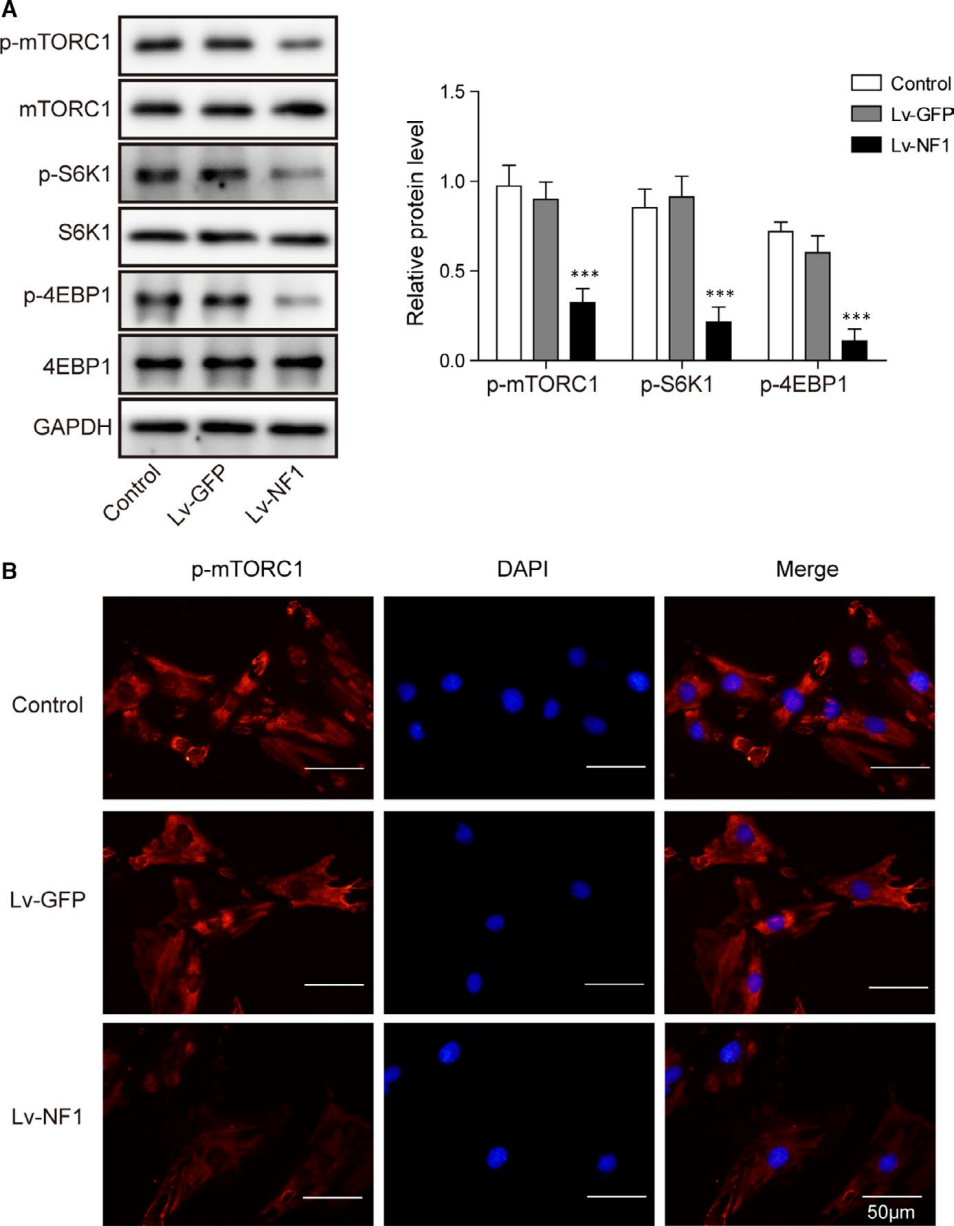
*NF1* inhibits mTORC1 signalling. A, Western blot of the phosphorylated mTORC1, S6K1, 4EBP1 and their references in Lv‐*NF1*, Lv‐GFP and control BMSCs. B, Immunostaining of p‐mTORC1 in Lv‐*NF1*, Lv‐GFP and control BMSCs. All data were presented as mean ± SD. ***P < .001. n = 6 in each group

To investigate the impact of the *NF1* gene on mTORC1 signalling pathway, we evaluated mTORC1‐related protein expressions by Western blotting and immunostaining. Western blotting data showed overexpression of *NF1* inhibited the phosphorylation of mTORC1, S6K1 and 4EBP1 (Figure 3A). Moreover, immunostaining of p‐mTORC1 confirmed that *NF1* overexpression suppressed phosphorylation of mTORC1 (Figure 3B). Therefore, the possible mechanism of *NF1* promoting autophagy is via mTORC1 signalling inhibition.

### 3.4 mTORC1 signalling impacts NF1‐mediated BMSCs osteogenic differentiation

**FIGURE 4:**
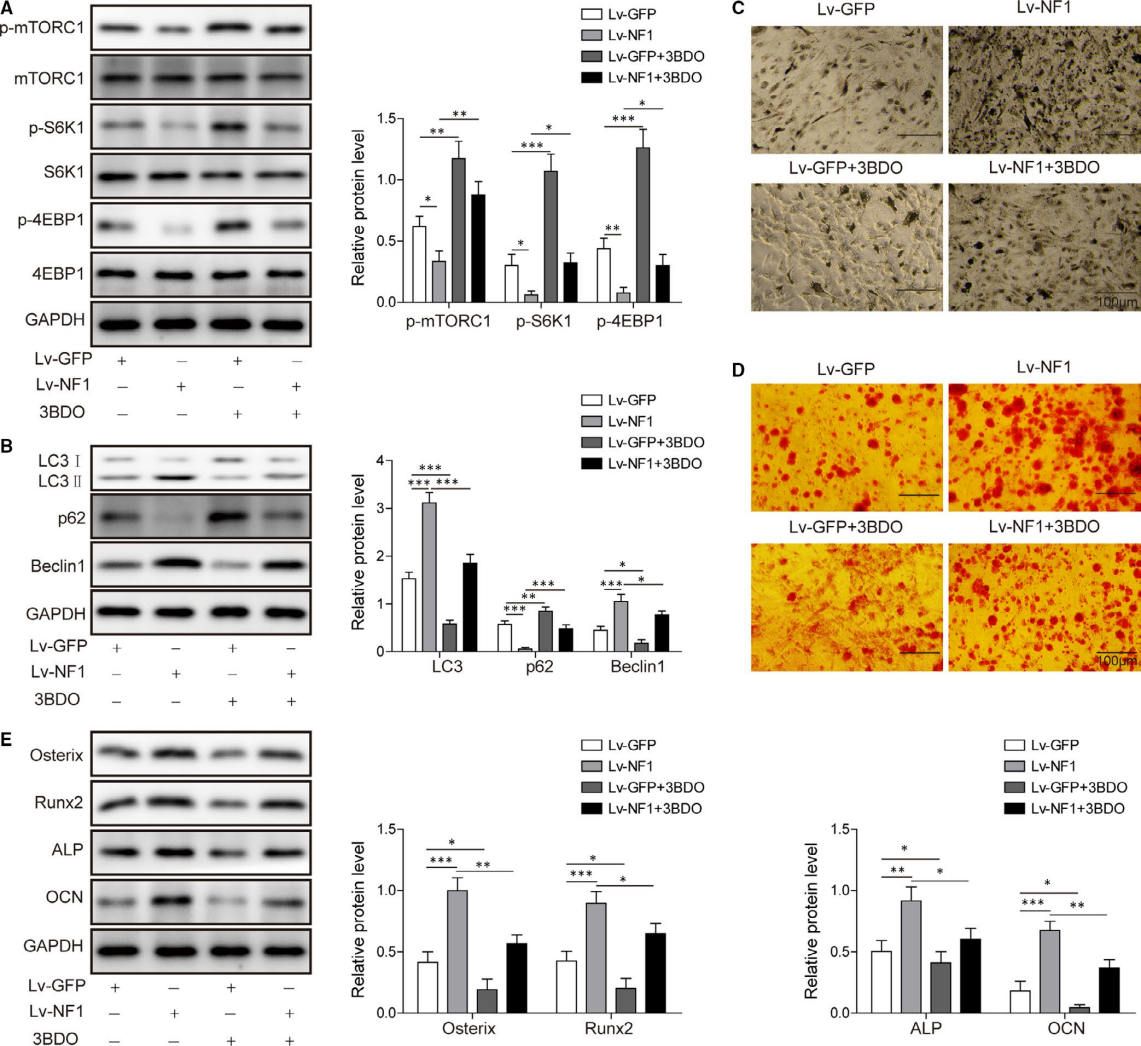
mTORC1 activation inhibits *NF1*‐mediated BMSCs differentiation. A, Western blot of phosphorylated mTORC1, S6K1, 4EBP1 and their references in Lv‐GFP, Lv‐*NF1*, Lv‐GFP + 3BDO and Lv‐*NF1* + 3BDO groups. B, Autophagy‐related protein LC3 II/I, p62 and Beclin1 levels in Lv‐GFP, Lv‐*NF1*, Lv‐GFP + 3BDO and Lv‐*NF1* + 3BDO groups. C, ALP and (D) Alizarin Red staining in Lv‐GFP, Lv‐*NF1*, Lv‐GFP + 3BDO and Lv‐*NF1* + 3BDO groups. E, Western blot of osteogenic markers Osterix, Runx2, ALP and OCN in Lv‐GFP, Lv‐*NF1*, Lv‐GFP + 3BDO and Lv‐*NF1* + 3BDO groups. All data were presented as mean ± SD. *P < .05; **P < .01; ***P < .001. n = 6 in each group

We next evaluated the role of mTORC1 signalling in *NF1*‐mediated BMSCs differentiation. Western blot showed Lv‐*NF1* group inhibited p‐mTORC1, S6K1 and 4EBP1 expressions, while activating mTOR by 3BDO significantly enhanced the p‐mTORC1, S6K1 and 4EBP1 expressions. When treating BMSCs with both *NF1* overexpression and 3BDO, the suppressive effect on mTORC1 signalling by *NF1* was weakened (Figure 4A). The autophagy activity was also impacted by mTOR activation and *NF1* overexpression. The *NF1* overexpression promoted autophagy activity by enhancing LC3 II/I and Beclin1 expressions and inhibiting p62 expression. Similar effect was achieved by using MEK inhibitor PD0325901 on BMSCs (Figure S1C). Lv‐GFP + 3BDO group inhibited autophagy activity by inhibiting LC3II/I and Beclin1 and enhancing p62. Treating *NF1* overexpressing cells with 3BDO attenuated Lv‐*NF1*‐activated autophagy (Figure 4B). ALP activity and cell mineralization were enhanced by *NF1* overexpression and suppressed by 3BDO (Figure 4C and D). Similar trend was seen in Western blotting data detecting osteogenic protein levels of Osterix, Runx2, ALP and OCN (Figure 4E). These results indicate mTORC1 signalling is involved in *NF1*‐mediated BMSCs osteogenic differentiation. Activation of mTORC1 pathway inhibits the osteogenic effect induced by *NF1* overexpression.

### 3.5 NF1 overexpression inhibits osteoclast differentiation

**FIGURE 5:**
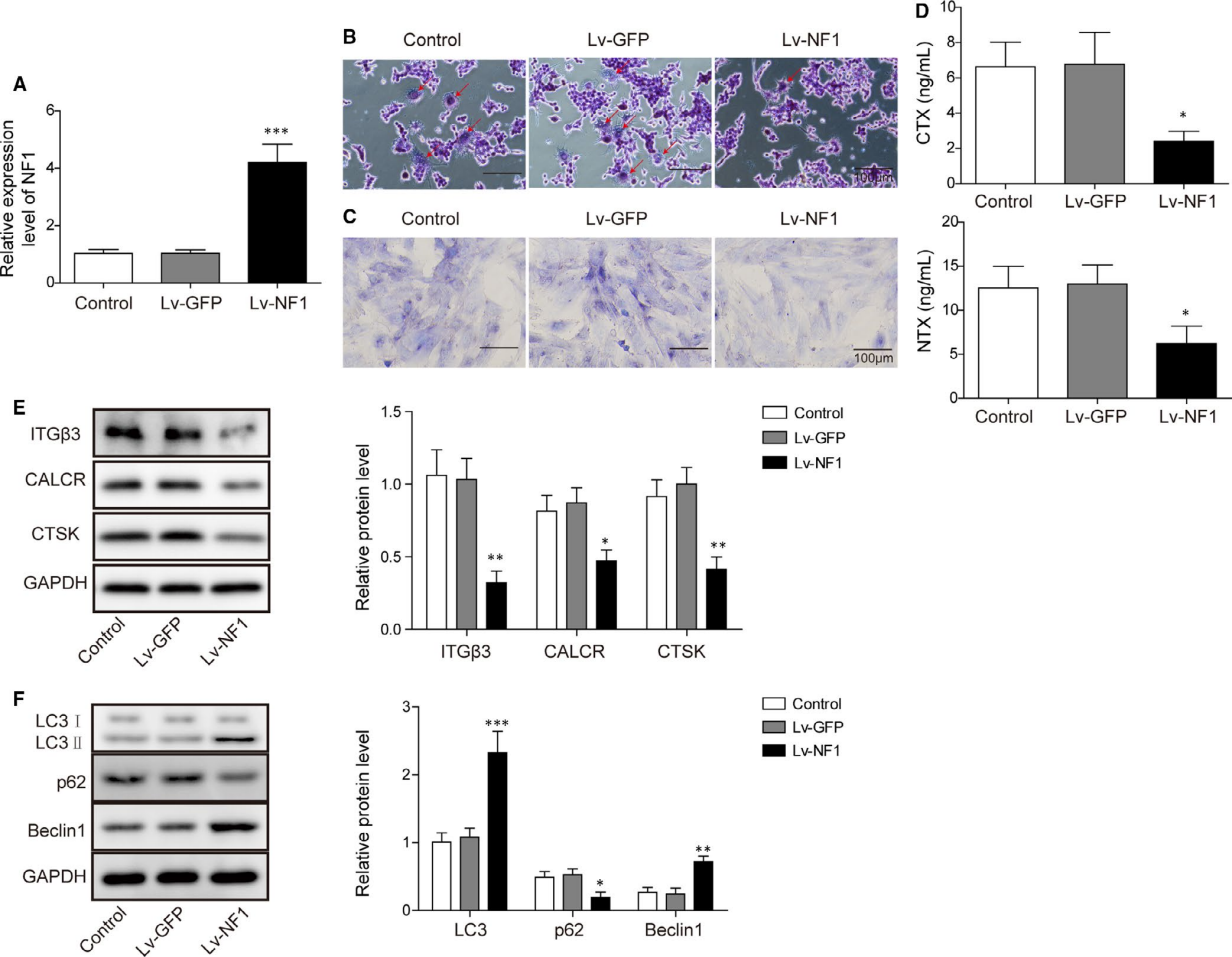
The *NF1* gene inhibits osteoclastic bone resorption. A, The *NF1* gene expression in control, Lv‐GFP and Lv‐*NF1* groups. B, TRAP staining to detect osteoclast differentiation in control, Lv‐GFP and Lv‐*NF1* groups. C, Bone resorption in control, Lv‐GFP and Lv‐*NF1* groups. D, ELISA of osteoclastic markers CTX and NTX levels in control, Lv‐GFP and Lv‐*NF1* groups. E, Western blot of osteoclastic markers ITGβ3, CALCR and CTSK in control, Lv‐GFP and Lv‐*NF1* groups. F, Western blot of autophagy‐related proteins in CTSK in control, Lv‐GFP and Lv‐NF1 groups. All data were presented as mean ± SD. *P < .05; **P < .01. n = 6 in each group

The efficacy of the *NF1* gene overexpression was validated by qRT‐PCR. Lv‐*NF1* group demonstrated significantly higher *NF1* gene expression in BM cells, indicating a successful transfection (Figure 5A). TRAP staining and bone resorption assay demonstrated inhibited osteoclast differentiation and less bone resorption in Lv‐*NF1* group in comparison with control and Lv‐GFP groups (Figure 5B and C). Bone metabolism markers CTX and NTX were also significantly reduced in Lv‐*NF1* group (Figure 5D). Western blotting data further demonstrated osteoclast‐related markers ITGβ3, CALCR and CTSK were reduced in Lv‐*NF1* group (Figure 5E). Autophagy activity was promoted by Lv‐*NF1*, as LC3II/I and Beclin1 expressions were enhanced, while p62 was inhibited in Lv‐*NF1* group (Figure 5F). Similar with Lv‐*NF1*, MEK inhibitor treatment also enhanced autophagy activity (Figure S1D). These results indicated *NF1* overexpression inhibits osteoclast differentiation.

### 3.6 NF1 promotes bone formation during fracture healing by enhancing autophagy via mTORC1 signalling

**FIGURE 6:**
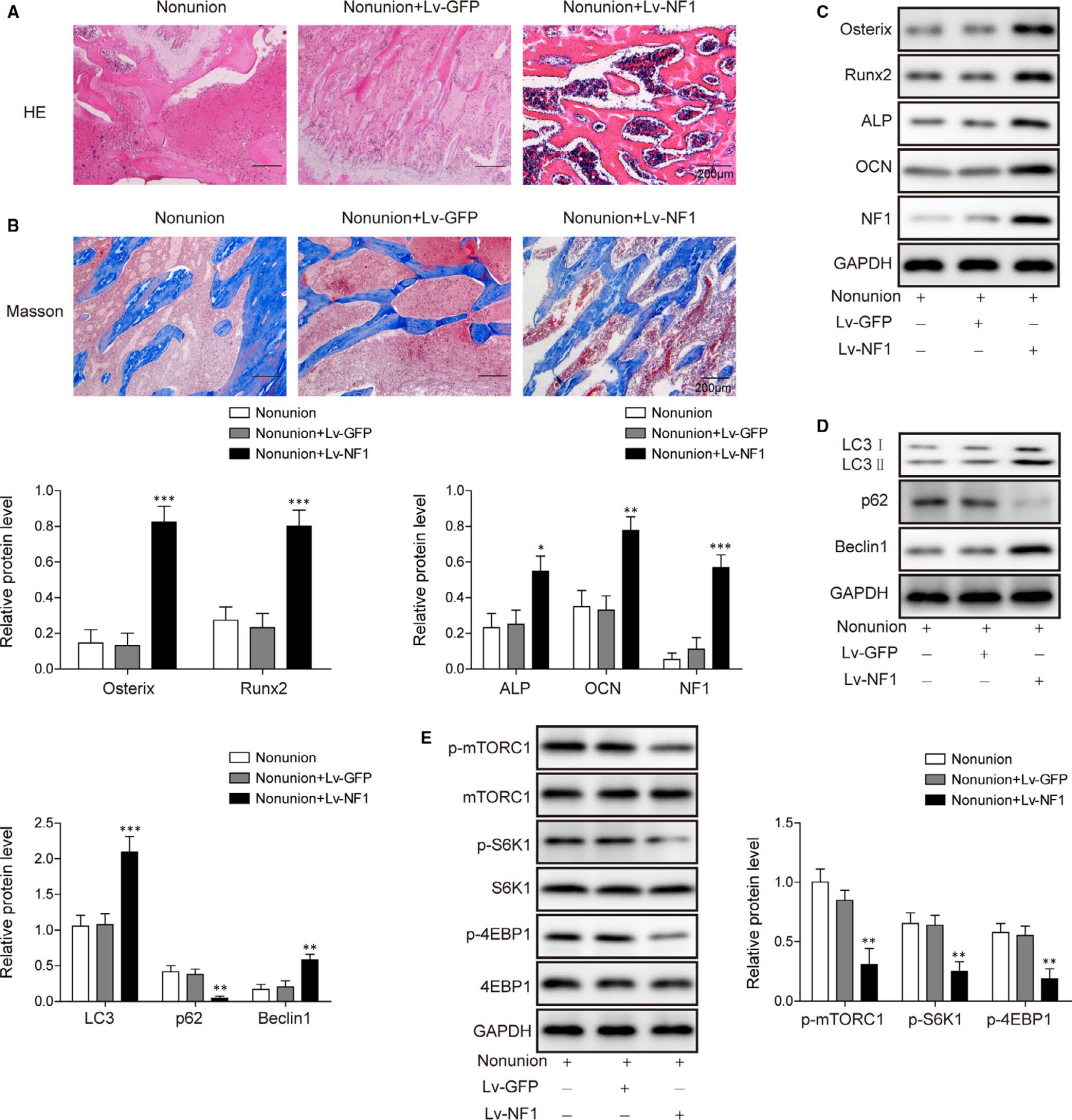
The *NF1* gene promotes bone formation in rat femur fracture model. A, H&E staining and (B) Masson's trichrome staining in Non‐union, Non‐union + Lv‐GFP and Non‐union + Lv‐*NF1* groups. Western blot to evaluated the protein levels of (C) osteogenic markers Osterix, Runx2, ALP and OCN, (D) autophagy makers LC3 I, II, p62 and Beclin1, and (E) mTORC1 signalling proteins in Non‐union, Non‐union + Lv‐GFP and Non‐union + Lv‐*NF1* groups. All data were presented as mean ± SD. *P < .05; **P < .01; ***P < .001. n = 6 in each group

With rat non‐union bone fracture model, we examined the effect of *NF1* overexpression to bone formation. H&E and Masson's trichrome staining presented histological evidences of newly formed bones. As shown in Figure 6A and B, more bony formation was observed in Lv‐*NF1* group, while control groups have less bony formation in the fracture region. Western blot data of osteogenic proteins Osterix, Runx2, ALP and OCN proved Lv‐*NF1* enhanced osteogenesis in rat non‐union bone fracture model (Figure 6C). Autophagy‐related marker LC3 II/I and Beclin1 was also enhanced by Lv‐*NF1* treatment, while p62 was suppressed in the meantime (Figure 6D). Western blotting data demonstrated p‐mTORC1, S6K1 and 4EBP1 were inhibited by Lv‐*NF1* treatment (Figure 6E), indicating mTORC1 inhibition played a role in *NF1*‐mediated osteogenesis. ERK activity was also inhibited by *NF1* overexpression, as p‐ERK expression was inhibited in Lv‐*NF1* group (Figure S1B). The data presented above suggest *NF1* overexpression enhances autophagy via inhibiting mTORC1 signalling, to promote bone formation during fracture healing.

## 4 DISCUSSION

Bone fracture healing is a complex biological process that requires the participation of complicated biochemical markers and signalling pathways. Despite the bone is one of the few tissues with high regenerative capacity, the healing process sometimes fails with the development of non‐union.^13^ In the present study, we evaluated the *NF1* gene expression in normal healing bones and non‐union bones, and found down‐regulated *NF1* expression in the non‐union bones. Overexpressing the *NF1* gene enhanced autophagy and osteogenic differentiation in BMSCs, and suppressed osteoclastic differentiation in BM cells via mediating mTORC1 signalling. Out results for the first time reveal the potential mechanism of the modulatory role of *NF1* in the bone formation during fracture healing process.

The *NF1* gene encodes neurofibromin 1 that regulates RAS/MAPK pathway. It has relatively high mutation rate, and the mutation results in alteration of cell growth and neural development. Previous study conducted by Kuorilehto et al associated the *NF1* gene expression with mouse fracture healing and rat pseudarthrosis,^14^ and reported normal *NF1* function was needed to achieve a normal bone fracture healing. When *NF1* function is impaired, reduced osteogenic activity is seen in *NF1*‐deficient model.^15^ In our current study, we found reduced *NF1* expression in non‐union bones as compared to normal fracture bones, supporting the theory that *NF1* is critical in bone fracture healing process. Autophagy is the process that allows cells to recycle damaged cellular components, and it plays an essential role in multiple tissue functions such as liver^16^ and bone,^17^ as well as in diseases such as cancers.^18^ In skeletal tissue regeneration process, studies have shown autophagy promotes osteogenic differentiation in BMSCs,^19^ and impairment of autophagy may cause abnormal ageing in skeletal tissues.^20^ Although the *NF1* gene and autophagy activity have both demonstrated their critical roles in skeletal tissue regeneration, the regulatory relationship between the *NF1* gene and autophagy is still unclear. Our present study demonstrated *NF1* overexpression significantly promoted autophagy activity, leading to enhanced osteogenic differentiation of BMSCs and inhibited osteoclastic differentiation in BM cells. Moreover, we also proved when treating cells with *NF1* overexpression and autophagy inhibitor, the osteogenic effect induced by *NF1* overexpression was partially inhibited, indicating autophagy activity was influencing the *NF1*/osteogenesis axis. One of the important signalling pathways that modulates cell autophagy is mTOR pathway. mTOR is a master regulator of cellular metabolism that has critical functions in cell growth and proliferation. Previous researchers have revealed the important role of mTOR in regulating cell autophagy. Zhang et al^21^ reported depleting of mTOR up‐regulated autophagy and inhibited mice osteoarthritis development. Cheng et al^22^ revealed AMPK/mTOR signalling pathway is responsible in activating autophagy promote osteogenic differentiation. In addition to regulating autophagy, mTOR pathway is also essential in *NF1* gene modulated cellular functions. Carnes et al^23^ stated loos of *NF1* gene lead to an increase in mTOR pathway. Li et al^24^ who studied BMSCs differentiation demonstrated mTOR’s important role in *NF1* modulated osteogenic differentiation. The Ras/MAPK/ERK signalling and mTOR signalling are critical mechanisms for mediating cell survival and differentiation. The results from the current study proved as the key regulator of Ras/MAPK pathway, *NF1* overexpression inhibited ERK and mTOR signalling, leading to an enhanced autophagy activity to promote osteogenesis. Our study for the first time associated the *NF1* gene expression with mTORC1 signalling and autophagy activity, revealing the potential molecular mechanism of the *NF1*‐regulated osteogenesis. The detailed regulatory mechanism will be investigated in our future study to establish the signalling axis.

Osteoclastic bone resorption is essential in bone remodelling. However, overactivated osteoclastic bone resorption may cause excessive bone loss and lead to delayed new bone formation and osteoporosis,^25^ which is one of the symptoms of *NF1* mutation‐related disease neurofibromatosis type 1.^26^ Yang et al^27^ showed an increased osteoclast activity in neurofibromatosis type 1 mice, and Ghadakzadeh et al^28^ also reported the hyperactivity of osteoclasts in *NF1*‐deficient mice, and by inhibiting osteoclast activity with β‐Catenin knockout, enhanced bone repair was achieved. In our study, we found overexpressing the *NF1* gene could effectively suppress osteoclastic bone resorption while enhancing osteoblastic differentiation and mineralization in the same time. This synergetic effect promotes bony formation in the fracture site in rat femur fracture model.

In conclusion, our study demonstrated overexpression of the *NF1* gene promoted osteogenic differentiation and bone formation during fracture by enhancing cell autophagy via suppressing mTORC1 signalling pathway. This regulatory role of *NF1* may provide a therapeutic clue to improve bone fracture healing in clinical practice.

## CONFLICT OF INTEREST

The authors declare no conflict of interests.

## AUTHOR CONTRIBUTIONS

**Qian Tan:** Resources (lead); Writing‐review & editing (supporting). **Jiang‐Yan Wu:** Writing‐review & editing (supporting). **Yao‐Xi Liu:** Formal analysis (equal). **Kun Liu:** Writing‐review & editing (equal). **Jin Tang:** Formal analysis (equal); Writing‐review & editing (equal). **Wei‐Hua Ye:** Writing‐review & editing (supporting). **Guanghui Zhu:** Formal analysis (equal); Writing‐review & editing (supporting). **Haibo Mei:** Conceptualization (equal); Writing‐review & editing (equal). **Ge Yang:** Conceptualization (equal); Writing‐review & editing (equal).

## Supporting information

Fig S1

## Data Availability

All data generated or analysed during this study are included in this published article [and its supplementary information files].
