# Supplementary figures and images for: The neurofibromatosis type I gene promotes autophagy via mTORC1 signalling pathway to enhance new bone formation after fracture
Source: J Cell Mol Med. 2020 Aug 30;24(19):11524–34. doi: 10.1111/jcmm.15767 (PMC7576311; doi:10.1111/jcmm.15767)

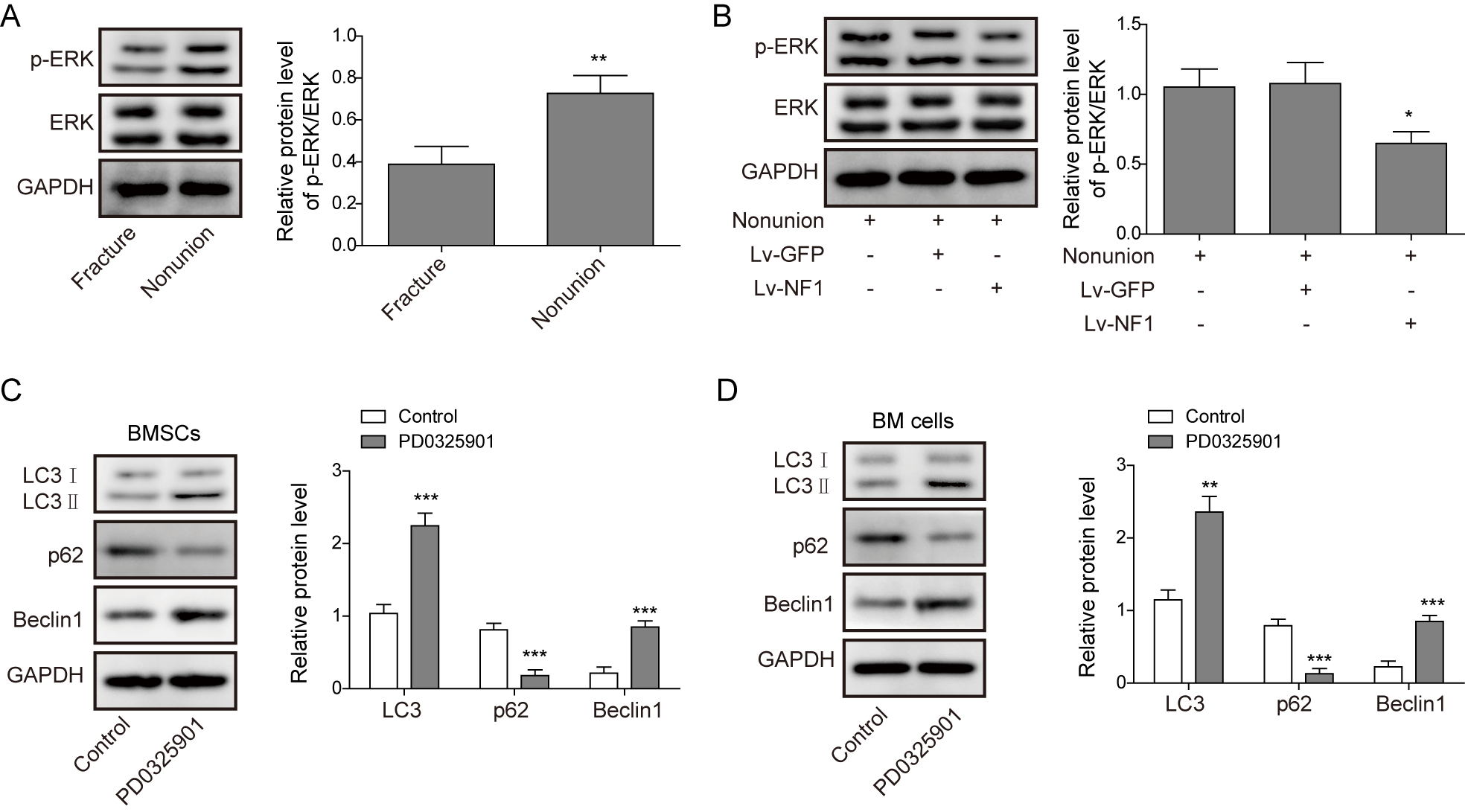

Supplement: Supplementary file 1 — Fig S1 [file JCMM-24-11524-s001.tif]
